# Supplementary material for: Epigenetic responses in Borrelia-infected Ixodes scapularis ticks: Over-expression of euchromatic histone lysine methyltransferase 2 and no change in DNA methylation
Source: PLoS One. 2025 Jun 5;20(6):e0324546. doi: 10.1371/journal.pone.0324546 (PMC12140222; doi:10.1371/journal.pone.0324546)
Supplement: S1 Tables — (DOCX) [file pone.0324546.s001.docx]

**Supplemental Table 1.** qPCR results for control housekeeping genes *l13a* and *rps4*

| Sample | Pathogen Infection Status | *l13a* Dup 1 Ct | *l13a* Dup 2 Ct | *l13a* Avg. Ct | *rps4* Dup 1 Ct | *rps4* Dup 2 Ct | *rps4* Avg. Ct |
| --- | --- | --- | --- | --- | --- | --- | --- |
| NS021 | Negative | 20.7 | 20 | 20.35 | 26.5 | 26.7 | 26.1 |
| NS026 | Negative | 23.2 | 23 | 23.1 | 30.1 | 30.2 | 30.15 |
| NS034 | Negative | 23.6 | 22.9 | 23.25 | 30.6 | 30.2 | 30.4 |
| NS035 | Negative | 25.1 | 26.1 | 25.6 | 33.4 | 34.5 | 33.95 |
| NS036 | Negative | 26.7 | 27.2 | 26.95 | 35.4 | 36.6 | 36 |
| NS037 | Negative | 20.7 | 20.7 | 20.7 | 26.7 | 26.9 | 26.8 |
| NS038 | Negative | 23.6 | 23.5 | 23.55 | 28 | 29.9 | 28.95 |
| NS041 | Negative | 21.9 | 22.2 | 22.05 | 27.8 | 27.9 | 27.85 |
| NS043 | Negative | 22.6 | 22.7 | 22.65 | 28.4 | 28.5 | 28.45 |
| NS048 | Negative | 23.3 | 24 | 23.65 | 31.1 | 30.2 | 30.65 |
| No DNA control^1, 2^ | N/A | N/A | N/A | N/A | N/A | N/A | N/A |
| No RNA control^3^ | N/A | N/A | N/A | N/A | N/A | N/A | N/A |

1. “N/A” means no Ct value was returned, suggesting no amplification. For calculations, this result was assigned a value of “40”.

2. “No DNA control” refers to a reaction without cDNA template input.

3. “No RNA control” refers to the cDNA reaction to which all reagents except RNA were added.

**Supplemental Table 2.** Nanodrop results for RNA extracted from *B. burgdorferi*-infected and -uninfected *I. scapularis* ticks

|  | | | | | | | |
| --- | --- | --- | --- | --- | --- | --- | --- |
| Sample ID | Infection  Status^1^ | ng/µL | A260 | A280 | 260/280 | 260/230 | Absorbance |
| NS015 | Positive | 68.5 | 1.711 | 1.259 | 1.36 | -0.72 | -2.372 |
| NS019 | Positive | 55.1 | 1.378 | 1.026 | 1.34 | -0.77 | -1.787 |
| NS031 | Positive | 63.9 | 1.598 | 1.149 | 1.391 | -0.77 | -2.071 |
| NS033 | Positive | 25.6 | 0.641 | 0.487 | 1.32 | -0.16 | -3.892 |
| NS040 | Positive | 21.5 | 0.538 | 0.404 | 1.33 | -0.21 | -2.557 |
| NS045 | Positive | 39.7 | 0.992 | 0.709 | 1.40 | -0.35 | -2.862 |
| NS047 | Positive | 44.4 | 1.110 | 0.759 | 1.46 | 0.18 | 6.332 |
| NS052 | Positive | 40.4 | 1.010 | 0.692 | 1.46 | 0.28 | 3.553 |
| NS084 | Positive | 27.9 | 0.689 | 0.470 | 1.49 | 0.27 | 2.627 |
| NS085 | Positive | 19.6 | 0.491 | 0.336 | 1.46 | 0.22 | 2.228 |
| NS021 | Negative | 19.6 | 0.491 | 0.323 | 1.52 | 0.38 | 1.308 |
| NS026 | Negative | 26.9 | 0.672 | 0.434 | 1.55 | 0.14 | 4.864 |
| NS034 | Negative | 37.0 | 0.925 | 0.649 | 1.43 | 0.37 | 2.525 |
| NS035 | Negative | 29.2 | 0.729 | 0.508 | 1.44 | 0.29 | 2.504 |
| NS036 | Negative | 58.3 | 1.457 | 1.062 | 1.37 | 0.51 | 2.871 |
| NS037 | Negative | 25.7 | 0.642 | 0.471 | 1.36 | 0.36 | 1.800 |
| NS038 | Negative | 28.7 | 0.718 | 0.513 | 1.40 | 0.17 | 4.208 |
| NS041 | Negative | 25.7 | 0.642 | 0.488 | 1.32 | -0.18 | -3.662 |
| NS043 | Negative | 32.8 | 0.819 | 0.591 | 1.39 | -0.29 | -2.870 |
| NS048 | Negative | 71.6 | 1.791 | 1.352 | 1.33 | -8.01 | -0.224 |
|  |  |  |  |  |  |  |  |

1. Infection status was determined by nested PCR

**Supplemental Table 3.** qPCR results for *Borrelia*-positive and -negative ticks with *EHMT2* and *l13a* primer sets

| Sample | Pathogen Infection Status1 | *EHMT2*-6 Dup 1 Ct | *EHMT2*-6 Dup | Avg. *EHMT2*-6 Ct | *EHMT2*-8 Dup | *EHMT2*-8 Dup 2 Ct | Avg *EHMT2*-8 Ct | *l13a* Dup 1 Ct | *l13a* Dup 2 Ct | Avg. |
| --- | --- | --- | --- | --- | --- | --- | --- | --- | --- | --- |
|  |  |  | 2 Ct |  | 1 Ct |  |  |  |  | *l13a* Ct |
| NS015 | Positive | N/A^2^ | 37.9 | N/A | N/A | 37.7 | N/A | 35.3 | 37.6 | 36.45 |
| NS019 | Positive | 35.2 | 34.6 | 34.9 | 38.4 | 35 | 36.7 | 31.6 | 32.5 | 32.05 |
| NS031 | Positive | 34.6 | 34 | 34.3 | 33.7 | N/A | N/A | N/A | 30.7 | N/A |
| NS033 | Positive | 31.4 | 31 | 31.2 | N/A | 35.3 | N/A | 28.3 | 29.4 | 28.85 |
| NS040 | Positive | 35.1 | 34.6 | 34.85 | 32.2 | N/A | N/A | 32.2 | 31.7 | 31.95 |
| NS045 | Positive | 34.8 | 33.8 | 34.3 | 36.3 | N/A | N/A | 30.5 | 30 | 30.25 |
| NS047 | Positive | 34.6 | 34.9 | 34.75 | 36.8 | 34.5 | 35.65 | 31.7 | 32.2 | 31.95 |
| NS052 | Positive | 34.9 | 33.3 | 34.1 | N/A | N/A | N/A | 29.2 | 30.8 | 35 |
| NS084 | Positive | 35.4 | N/A | N/A | 36.8 | 37.5 | 37.15 | 28.8 | 28.8 | 28.8 |
| NS085 | Positive | 33.7 | 31.3 | 32.5 | 32.7 | N/A | N/A | 27.8 | 25.9 | 26.85 |
| No DNA^3^ control | N/A | 34.5 | N/A | N/A | N/A | N/A | N/A | N/A | N/A | N/A |
| No RNA^4^ control | N/A | N/A | N/A | N/A | N/A | N/A | N/A | N/A | N/A | N/A |
| NS021 | Negative | 35.1 | 31.8 | 33.45 | 36.1 | 35.1 | 35.6 | 20.7 | 20 | 20.35 |
| NS026 | Negative | 30.9 | 30.4 | 30.65 | N/A | 36.4 | N/A | 23.2 | 23 | 23.1 |
| NS034 | Negative | 32.7 | 30.8 | 31.75 | 37.1 | 37.7 | 37.4 | 23.6 | 22.9 | 23.25 |
| NS035 | Negative | 34 | 33.1 | 33.55 | 37.3 | 38.2 | 37.75 | 25.1 | 26.1 | 25.6 |
| NS036 | Negative | 35.7 | 34.1 | 34.9 | N/A | N/A | N/A | 26.7 | 27.2 | 26.95 |
| NS037 | Negative | 29.5 | 28.6 | 29.1 | 36.7 | 36 | 36.35 | 20.7 | 20.7 | 20.7 |
| NS038 | Negative | 32.6 | 37.9 | 35.25 | 37.4 | 35.5 | 36.45 | 23.6 | 23.5 | 23.55 |
| NS041 | Negative | 36.8 | 37.1 | 36.95 | 37.9 | 36.6 | 37.25 | 21.9 | 22.2 | 22.05 |
| NS043 | Negative | N/A | 34.7 | N/A | N/A | 37.9 | N/A | 22.6 | 22.7 | 22.65 |
| NS048 | Negative | 37.4 | 31.3 | 34.35 | 33.3 | 36.5 | 34.9 | 23.3 | 24 | 23.65 |
| No DNA control^3^ | N/A | N/A | N/A | N/A | N/A | N/A | N/A | N/A | N/A | N/A |
| No RNA control^4^ | N/A | N/A | N/A | N/A | N/A | N/A | N/A | N/A | N/A | N/A |

1. Pathogen infection status determined by nested PCR.
2. N/A indicates that no Ct value was obtained, suggesting no amplification occurred. Note: “N/A” means no Ct value was returned, suggesting no amplification. For calculations, this result was assigned a value of “40”.
3. “No DNA control” refers to a reaction without cDNA input.
4. “No RNA control” refers to the cDNA reaction to which all reagents except RNA were added. qPCR results indicated some anomalies in qPCR readings, but these were not consistent across primers and no banding was present on gels after gel electrophoresis with *EHMT2-6* and *l13a* primers (Supplemental Table 6), so all samples were included in the study.

**Supplemental Table 4.** No Reverse Transcriptase (RT) controls

| Sample | Infection Status^1^ | *EHMT2*-6 Dup 1 Ct | *EHMT2*-6 Dup 2 Ct | Avg *EHMT2*-6 Ct | *EHMT2*-8 Dup 1 Ct | *EHMT2*-8 Dup 2 Ct | Avg. *EHMT2*-8 Ct | *l13a* Dup 1 Ct | *l13a* Dup 2 Ct | Avg. |
| --- | --- | --- | --- | --- | --- | --- | --- | --- | --- | --- |
|  |  |  |  |  |  |  |  |  |  | *l13a* Ct |
| NS015 | Positive | N/A^2^ | N/A | N/A | N/A | N/A | N/A | N/A | 37.6 | N/A |
| NS019 | Positive | 33.7 | N/A | N/A | N/A | N/A | N/A | N/A | N/A | N/A |
| NS031 | Positive | 34.5 | 36.1 | 35.31 | N/A | N/A | N/A | N/A | N/A | N/A |
| NS033 | Positive | N/A | N/A | N/A | N/A | N/A | N/A | N/A | N/A | N/A |
| NS040 | Positive | N/A | N/A | N/A | N/A | N/A | N/A | N/A | N/A | N/A |
| NS045 | Positive | 38 | 37.2 | 37.6 | N/A | 35.3 | N/A | N/A | 37.6 | N/A |
| NS047 | Positive | N/A | N/A | N/A | N/A | N/A | N/A | 35 | 35.1 | 35 |
| NS052 | Positive | N/A | N/A | N/A | N/A | 35.4 | N/A | N/A | 35.4 | N/A |
| NS084 | Positive | N/A | N/A | N/A | N/A | N/A | N/A | N/A | N/A | N/A |
| NS085 | Positive | N/A | N/A | N/A | N/A | N/A | N/A | N/A | N/A | N/A |
| No DNA control^3^ | N/A | N/A | N/A | N/A | N/A | N/A | N/A | N/A | N/A | N/A |
| No RNA control^4^ | N/A | N/A | N/A | N/A | N/A | N/A | N/A | N/A | N/A | N/A |
|  |  |  |  |  |  |  |  |  |  |  |
| NS021 | Negative | 38.5 | N/A | N/A | N/A | N/A | N/A | N/A | N/A | N/A |
| NS026 | Negative | N/A | N/A | N/A | N/A | N/A | N/A | N/A | N/A | N/A |
| NS034 | Negative | N/A | N/A | N/A | N/A | N/A | N/A | N/A | N/A | N/A |
| NS035 | Negative | N/A | N/A | N/A | N/A | N/A | N/A | N/A | N/A | N/A |
| NS036 | Negative | N/A | N/A | N/A | N/A | N/A | N/A | N/A | N/A | N/A |
| NS037 | Negative | N/A | N/A | N/A | N/A | N/A | N/A | N/A | N/A | N/A |
| NS038 | Negative | 31.4 | 31 | 31.2 | 35.8 | 35.8 | 35.8 | 28.3 | 28.9 | 28.6 |
| NS041 | Negative | N/A | N/A | N/A | N/A | N/A | N/A | N/A | N/A | N/A |
| NS043 | Negative | N/A | N/A | N/A | N/A | N/A | N/A | N/A | N/A | N/A |
| NS048 | Negative | N/A | N/A | N/A | N/A | N/A | N/A | N/A | N/A | N/A |
| No DNA control^3^ | N/A | N/A | N/A | N/A | N/A | N/A | N/A | N/A | N/A | N/A |
| No RNA control^4^ | N/A | N/A | N/A | N/A | N/A | N/A | N/A | N/A | N/A | N/A |

1. Infection status was determined by nested PCR.
2. N/A indicates that no Ct value was obtained, suggesting no amplification occurred. For calculations, this result was assigned a value of 40.
3. “No DNA control” refers to a reaction without cDNA input.
4. “No RNA control” refers to the cDNA reaction to which all reagents except RNA were added. qPCR results indicated some anomalies in qPCR readings, but these were not consistent across primers and no banding was present on gels after gel electrophoresis with *EHMT2-6* and *l13a* primers (Supplemental Table 6), so all samples were included in the study.
