# Supplementary material for: Epigenetic responses in Borrelia-infected Ixodes scapularis ticks: Over-expression of euchromatic histone lysine methyltransferase 2 and no change in DNA methylation
Source: PLoS One. 2025 Jun 5;20(6):e0324546. doi: 10.1371/journal.pone.0324546 (PMC12140222; doi:10.1371/journal.pone.0324546)
Supplement: S1 Fig — All primer optimizations steps were tested on tick DNA samples #590 and #592 from 2019. “– con” = no template negative control using water instead of DNA template. The 200–2000 base pair DNA ladder is to the left in each gel image. A: Gradient PCR products targeting the ISR1 region. B: Gradient PCR products targeting the ISR2C and ISR2D regions. C: Gradient PCR products targeting the ISR3 region. While primers ideally produce a single amplicon, as these are repeat regions primers ISR2C, ISR2D, and ISR3 produced multiple bands. (DOCX) [file pone.0324546.s002.docx]

**Supplemental Figure 1**


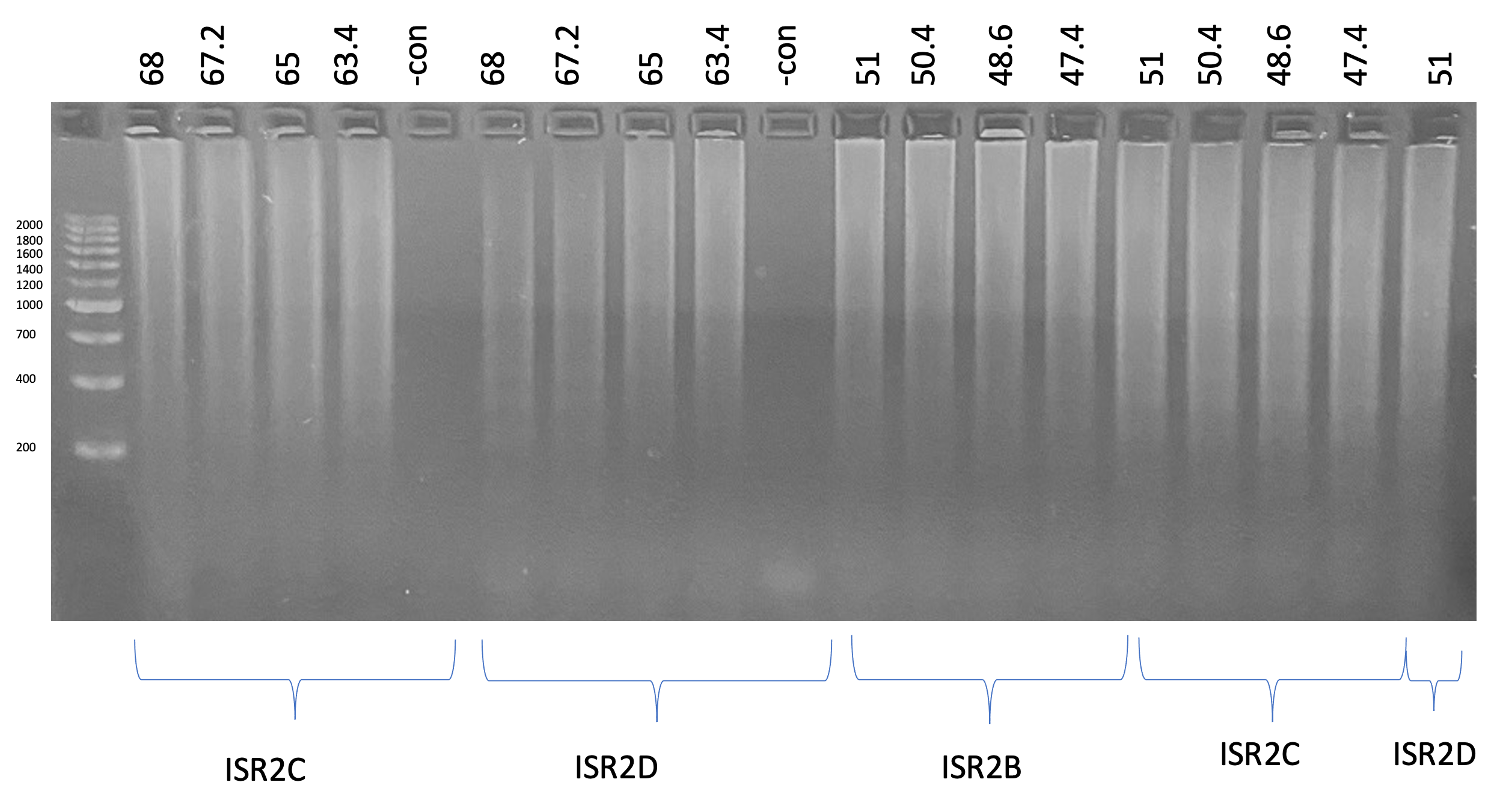


**A** **
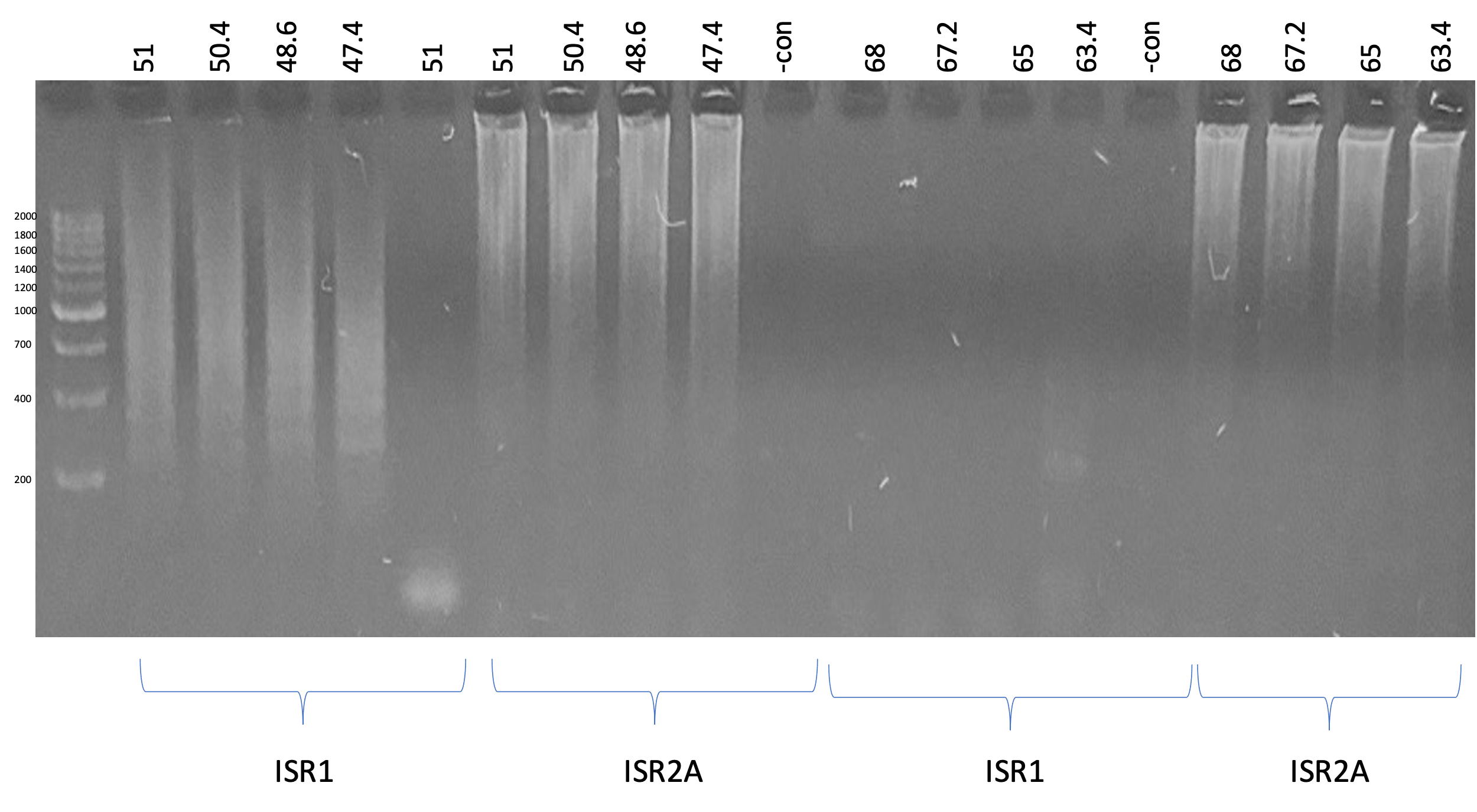
**

**B**

**
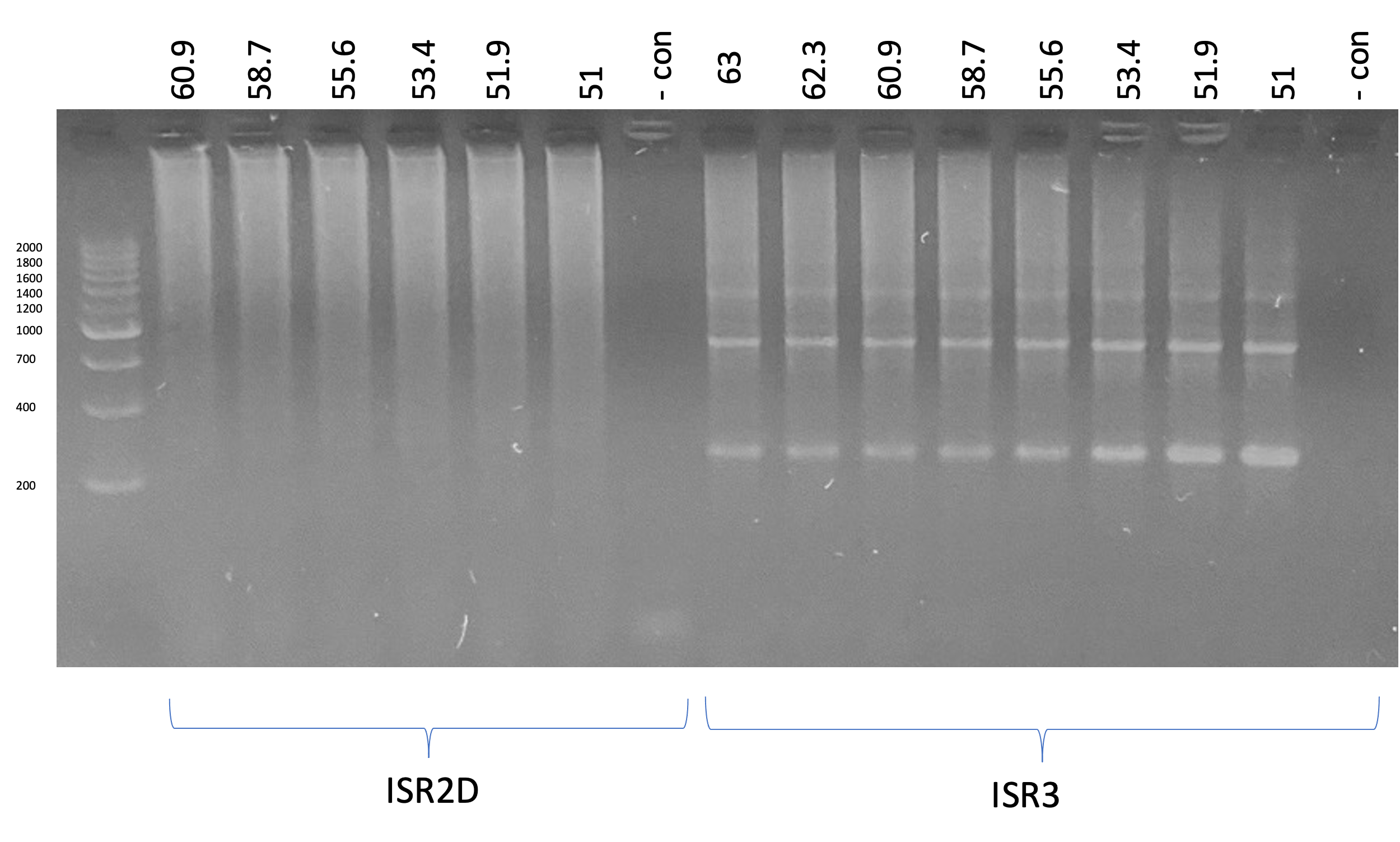
**

**C**

Supplemental Figure 1. Agarose gel of Gradient PCR products for ISR primers. All primer optimization steps were tested on tick DNA samples #590 and #592 from 2019. “– con” = no template negative control using water instead of DNA template. The 200-2000 base pair DNA ladder is to the left in each gel image. A: Gradient PCR products targeting the ISR1 and ISR2A regions. B: Gradient PCR products targeting the ISR2B, ISR2C and ISR2D regions. C: Gradient PCR products targeting the ISR2D and ISR3 regions. While primers ideally produce a single amplicon, these primers produced multiple bands or smears as these are repeat regions.
