# Supplementary material for: Epigenetic responses in Borrelia-infected Ixodes scapularis ticks: Over-expression of euchromatic histone lysine methyltransferase 2 and no change in DNA methylation
Source: PLoS One. 2025 Jun 5;20(6):e0324546. doi: 10.1371/journal.pone.0324546 (PMC12140222; doi:10.1371/journal.pone.0324546)
Supplement: S2 Fig — Gel electrophoresis of qPCR amplification of cDNA from samples NS021, 026, 034, 035, 037, 038, 041, 043, 048 negative ticks. A) rps4 primers, with an amplicon consistent with the predicted size of 80 bp B) l13a primers, with an amplicon consistent with the predicted size of ~280 bp. (DOCX) [file pone.0324546.s003.docx]

**Supplemental Figure 2**


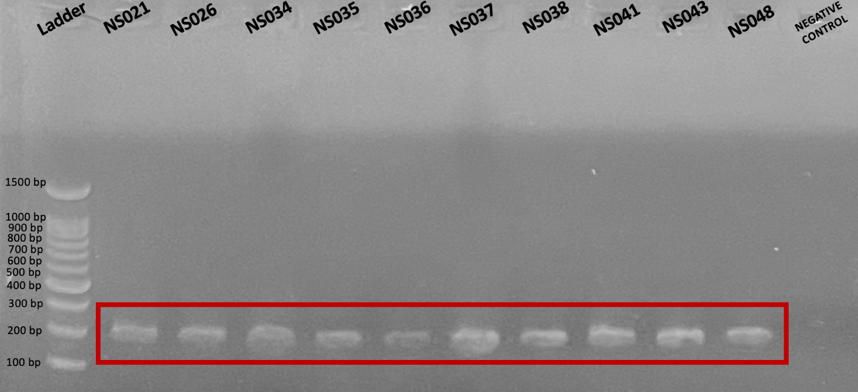

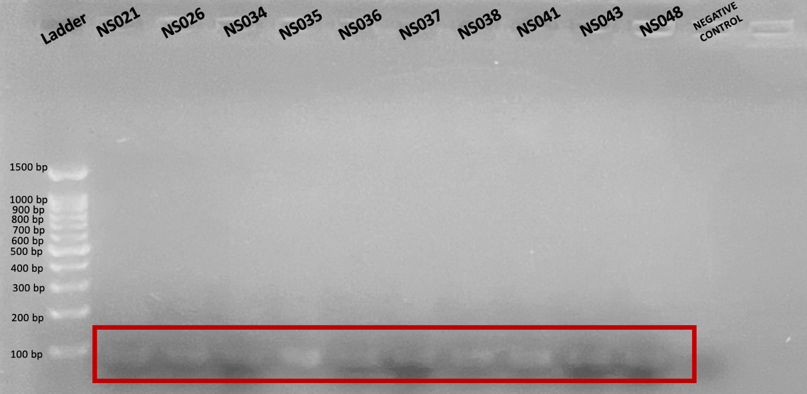


**A**

**B**

**Supplemental Figure 2**. Gel electrophoresis of qPCR amplification of cDNA from samples NS021, 026, 034, 035, 037, 038, 041, 043, 048 negative ticks. A) *rps4* primers, with an amplicon consistent with the predicted size of 80 bp B) *l13a* primers, with an amplicon consistent with the predicted size of ~280 bp.
