# Supplementary material for: Epigenetic responses in Borrelia-infected Ixodes scapularis ticks: Over-expression of euchromatic histone lysine methyltransferase 2 and no change in DNA methylation
Source: PLoS One. 2025 Jun 5;20(6):e0324546. doi: 10.1371/journal.pone.0324546 (PMC12140222; doi:10.1371/journal.pone.0324546)
Supplement: S3 Fig — EHMT2 6 and l13a standard curves. A) Scatterplot with trendline for a three series dilution of EHMT2 6 primers. The slope of the trendline was calculated to be −2.175, with an R2 value of 0.8183. The amplification efficiency was thus calculated to be 1.88, or 188%. B) Scatterplot with trendline for a three series dilution of l13a. The slope of the trendline was calculated to be −2.15, with an R2 value of 0.9197. The amplification efficiency was thus calculated to be 1.92, or 192%. C) qPCR results. (DOCX) [file pone.0324546.s004.docx]

**Supplemental Figure 3**

| Dilution | Avg *EHMT2*-6 Ct | Avg *l13a* Ct | |
| --- | --- | --- | --- |
| 1 | 32.25 | 30 |  |
| 0.5 | 32.65 | 33.25 |  |
| 0.25 | 36.6 | 34.3 |  |
| 0.125 | 36.65 | 36.25 |  |
| 0.0625 | 37.25 | 38.65 |  |
| 0.0312 | 38.7 | N/A |  |
| 0.016 | N/A | N/A |  |
| 0.0078125 | 36.15 | 38.95 |  |
| 0.00390625 | 38.85 | N/A |  |
| 0 | 37.25 | N/A |  |

***
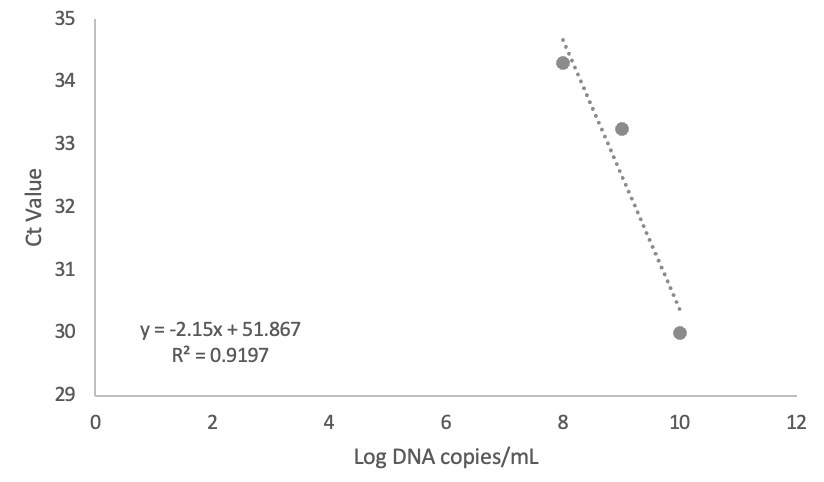

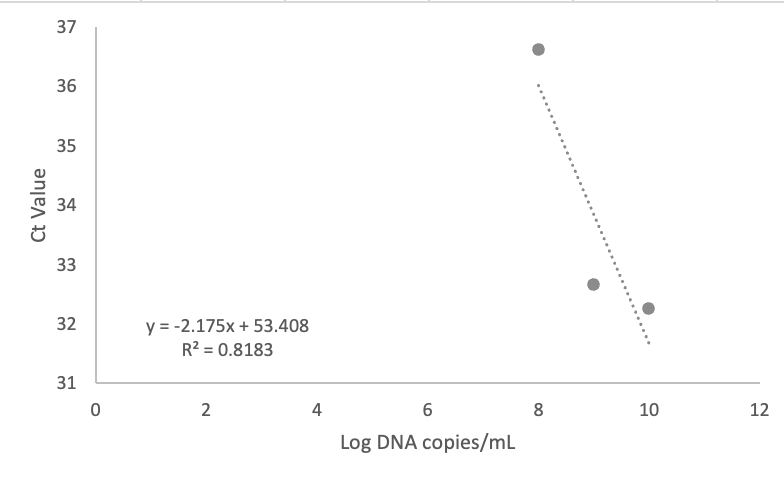
*A B. C.**

**Supplemental Figure 3**. *EHMT2*-6 and *l13a standard curves.* A) Scatterplot with trendline for a three series dilution of *EHMT2-6* primers. The slope of the trendline was calculated to be -2.175, with an R^2^ value of 0.8183. The amplification efficiency was thus calculated to be 1.88, or 188%. B) Scatterplot with trendline for a three series dilution of *l13a*. The slope of the trendline was calculated to be -2.15, with an R^2^ value of 0.9197. The amplification efficiency was thus calculated to be 1.92, or 192%. C) Average Ct values for the serial dilution of standards using *EHMT2* and *l13a* primer sets.
