# Supplementary material for: Epigenetic responses in Borrelia-infected Ixodes scapularis ticks: Over-expression of euchromatic histone lysine methyltransferase 2 and no change in DNA methylation
Source: PLoS One. 2025 Jun 5;20(6):e0324546. doi: 10.1371/journal.pone.0324546 (PMC12140222; doi:10.1371/journal.pone.0324546)
Supplement: S4 Fig — The 200–2000 bp ladder was used for A as a DNA size reference. A: Methylated and unmethylated spike-in control qPCR gel results. Water was used as the negative control. IP = immunoprecipitated, input = total input DNA. B: qPCR melt curve and amplification curve for the unmethylated spike-in control. C: qPCR melt curve and amplification curve for the methylated spike-in control. The well labels on the gel represent the identity of the tick with IP being immunoprecipitation and the input DNA from the tick samples. The methylated and unmethylated spike-in DNA were used corresponding with the methylated and unmethylated primers. (DOCX) [file pone.0324546.s005.docx]

**Supplemental Figure 4**

**A**


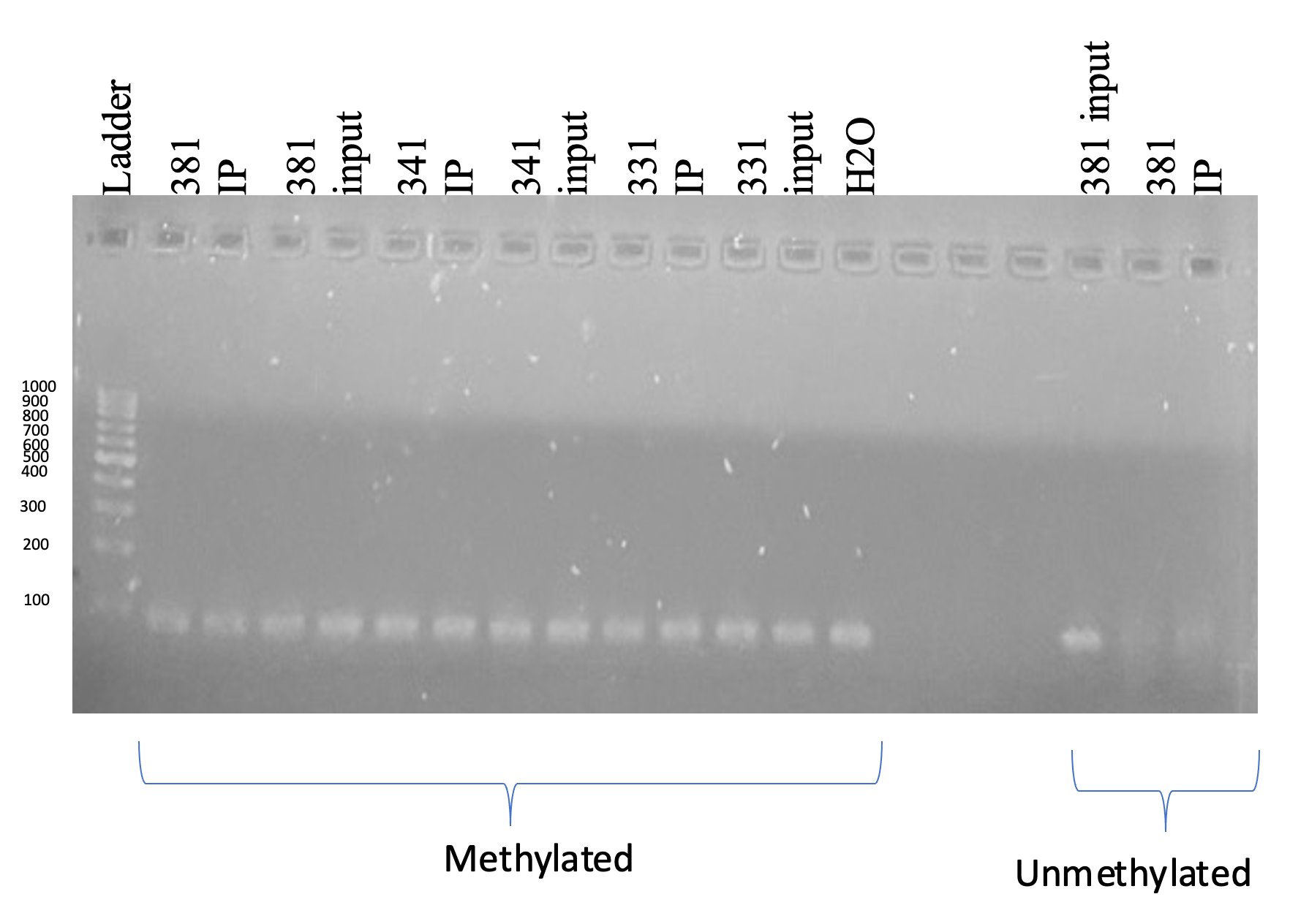


**B**


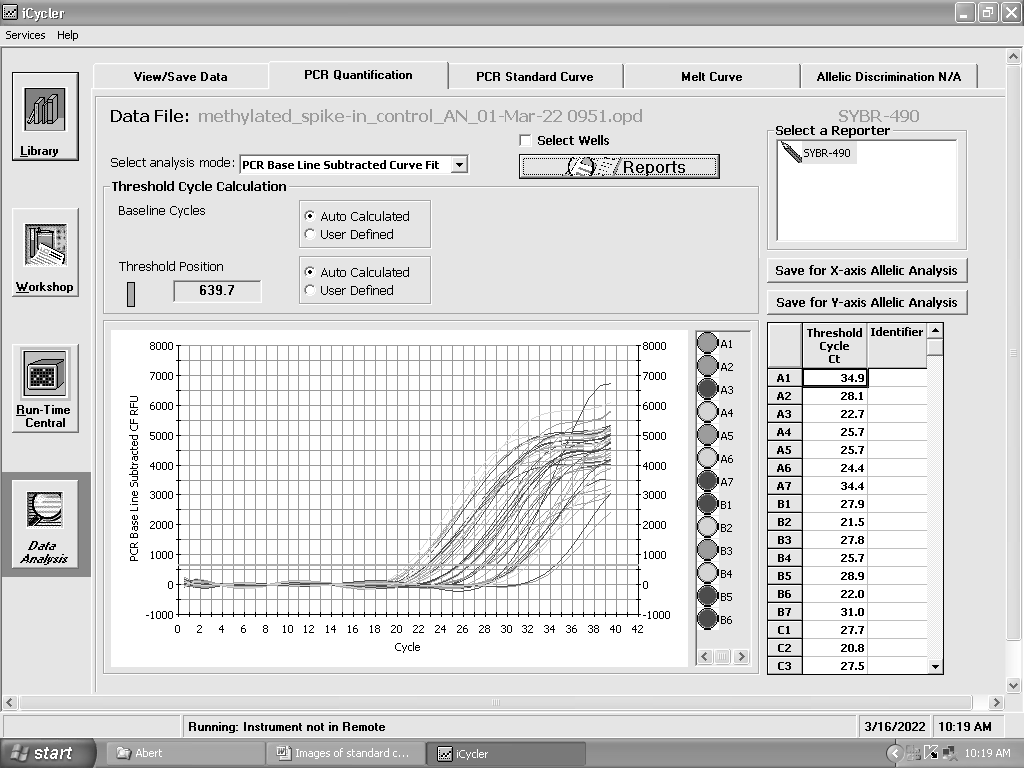

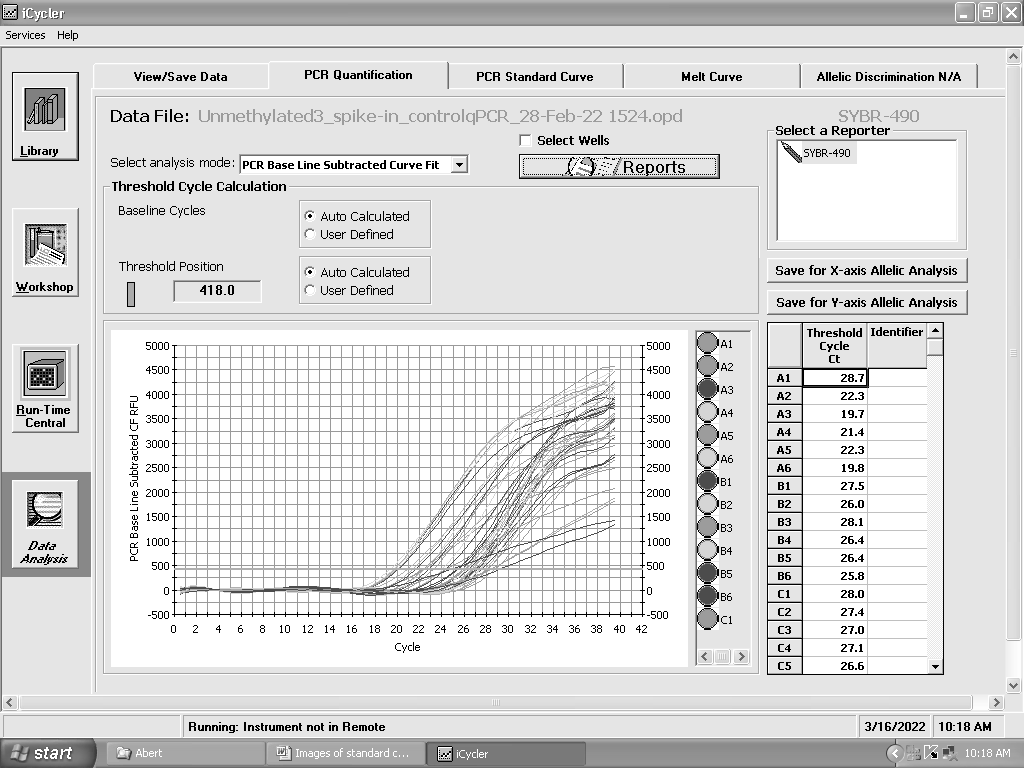
**C**
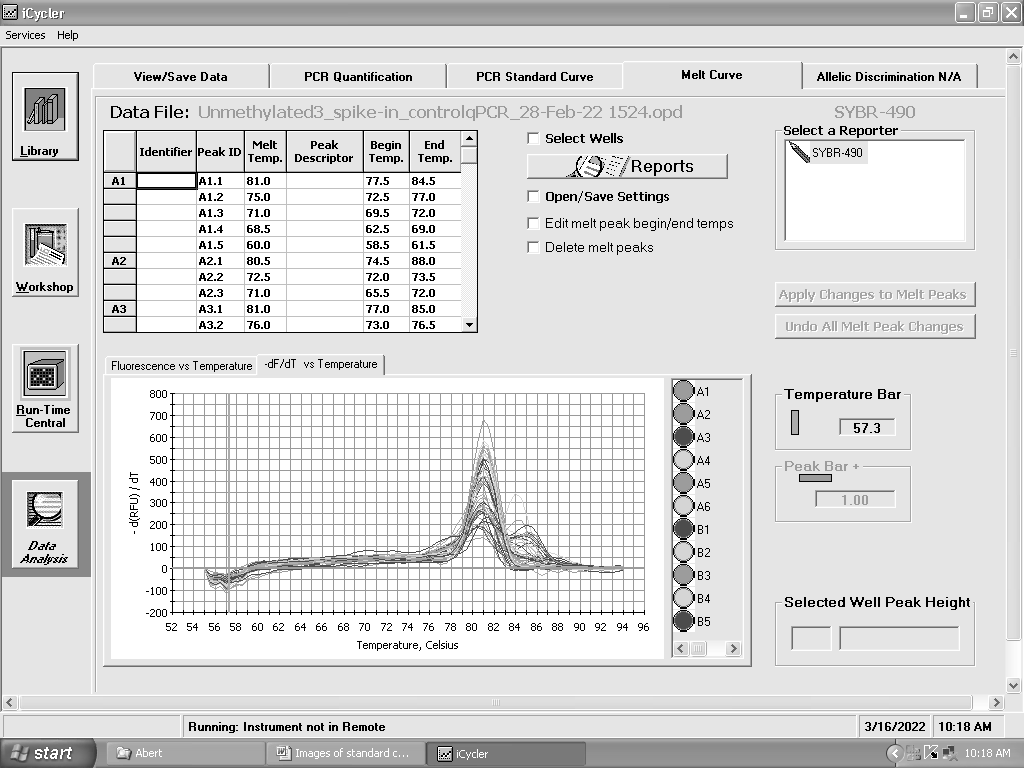


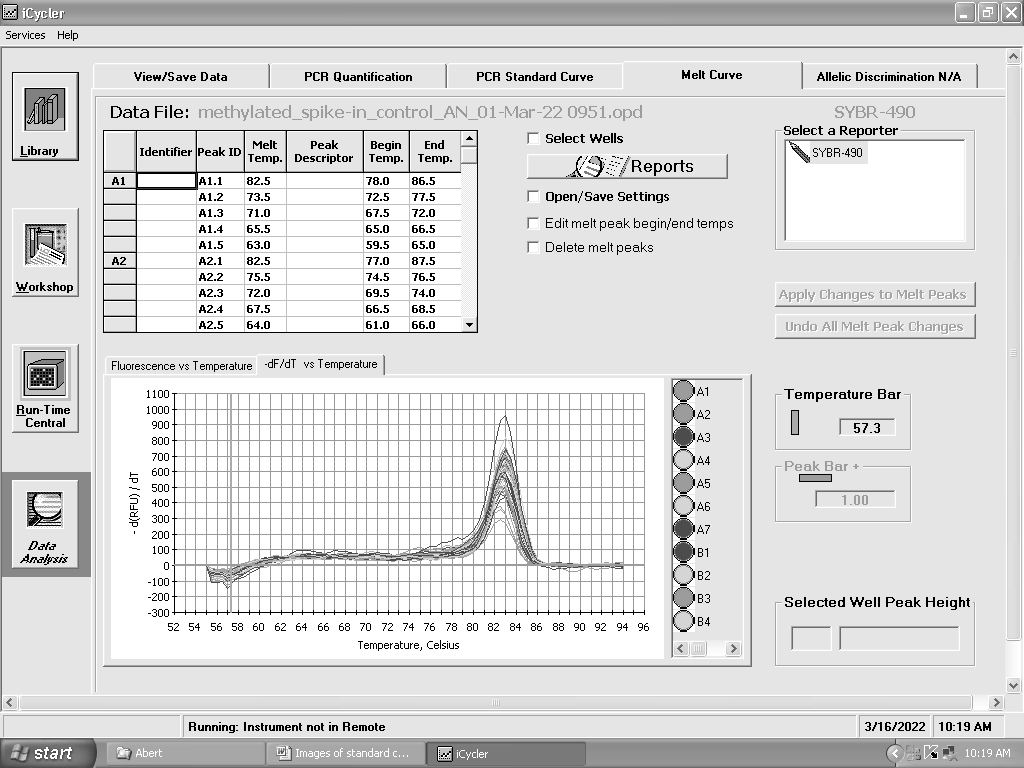


Supplemental Figure 4. Control qPCR reaction to determine MeDIP efficiency using the spike-in DNA controls and primers. The 200-2000 bp ladder was used for panel A as a DNA size reference. A: Methylated and unmethylated spike-in control qPCR gel results. Water was used as the negative control. IP = immunoprecipitated, input = total input DNA. B: qPCR melt curve and amplification curve for the unmethylated spike-in control. C: qPCR melt curve and amplification curve for the methylated spike-in control. The methylated and unmethylated spike-in DNA used corresponded with primers that detect methylated and unmethylated DNA.
