# Supplementary material for: Epigenetic responses in Borrelia-infected Ixodes scapularis ticks: Over-expression of euchromatic histone lysine methyltransferase 2 and no change in DNA methylation
Source: PLoS One. 2025 Jun 5;20(6):e0324546. doi: 10.1371/journal.pone.0324546 (PMC12140222; doi:10.1371/journal.pone.0324546)
Supplement: S5 Fig — B) Synthesized cDNA from each sample underwent qPCR with EHMT2 8 primers, with an amplicon size of 184 bp. C) Synthesized cDNA from each sample underwent qPCR with rps4 primers, with an amplicon size of 80 bp. D) Synthesized cDNA from each sample underwent qPCR with l13a primers, with an amplicon size of ~280 bp. (DOCX) [file pone.0324546.s006.docx]

**Supplemental Figure 5**


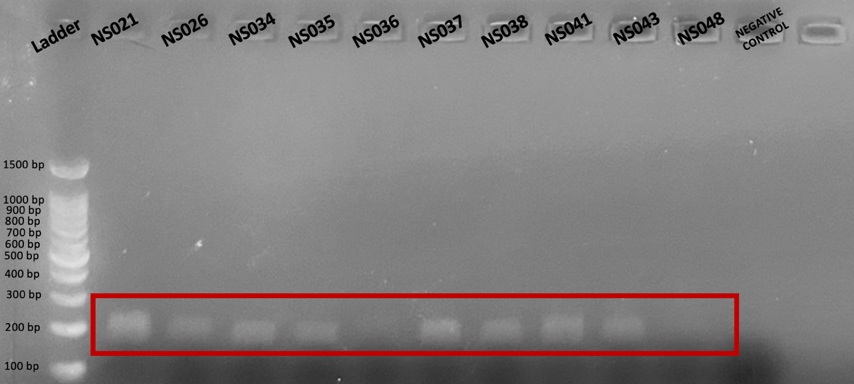

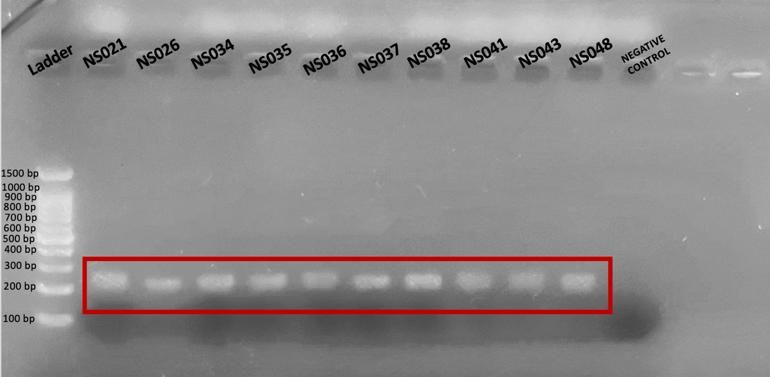


A

B


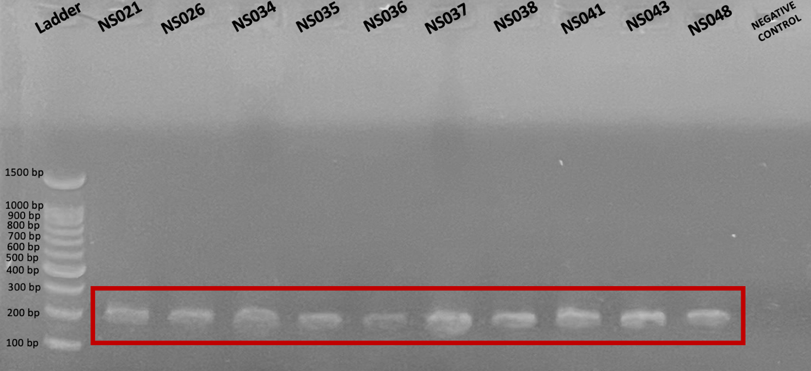

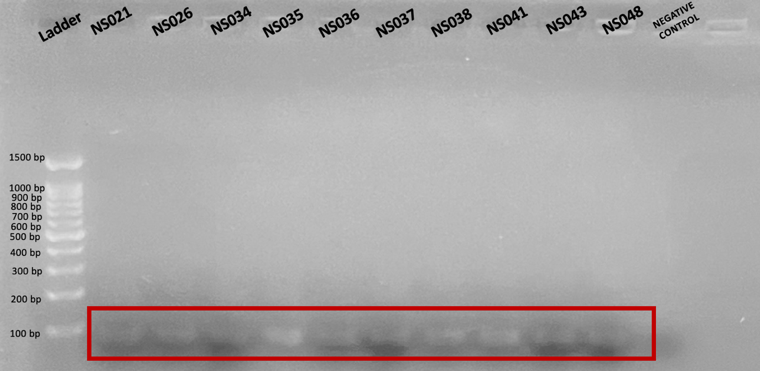


D

C

**Supplemental Figure 5.** Agarose gel electrophoresis of qPCR results. A: Synthesized cDNA from each sample underwent qPCR with *EHMT2*-6 primers, with an amplicon size of 207 bp. B: Synthesized cDNA from each sample underwent qPCR with *EHMT2*-8 primers, with an amplicon size of 184 bp. C: Synthesized cDNA from each sample underwent qPCR with *rps4* primers, with an amplicon size of 80 bp. D: Synthesized cDNA from each sample underwent qPCR with *l13a* primers, with an amplicon size of ~280 bp.
