# Supplementary material for: Epigenetic responses in Borrelia-infected Ixodes scapularis ticks: Over-expression of euchromatic histone lysine methyltransferase 2 and no change in DNA methylation
Source: PLoS One. 2025 Jun 5;20(6):e0324546. doi: 10.1371/journal.pone.0324546 (PMC12140222; doi:10.1371/journal.pone.0324546)
Supplement: S6 Fig — A) No-RT controls from cDNA synthesis for negative samples that underwent qPCR with EHMT2 6 primers, with an amplicon size of 207 bp. One sample, NS038 was found to contain residual genomic DNA (box), whereas the rest contained no genomic DNA. B) No-RT controls from cDNA synthesis for negative samples that underwent qPCR with EHMT2 8 primers, with an amplicon size of 184 bp. NS038 was again found to contain residual genomic DNA (box), whereas the rest contained no genomic DNA. C) No-RT controls from cDNA synthesis for each positive tick sample underwent qPCR with EHMT2 6 primers, with an amplicon size of 207 bp. D) No-RT controls from cDNA synthesis for each positive tick sample underwent qPCR with l13a primers, with an amplicon size of ~280 bp. The ladder goes from 100 bp increments to 1000 bp and the top band is 1,500pb. (DOCX) [file pone.0324546.s007.docx]

**Supplemental Figure 6**

**
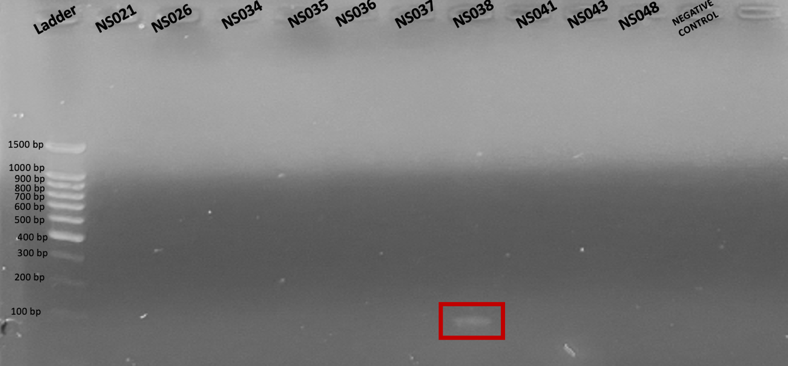

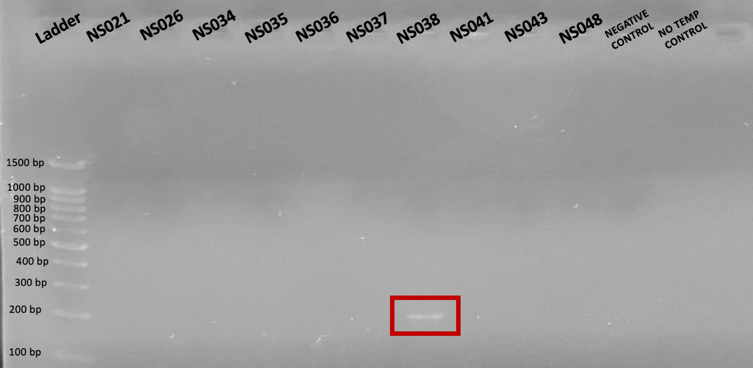
**

B

A

D

C

**
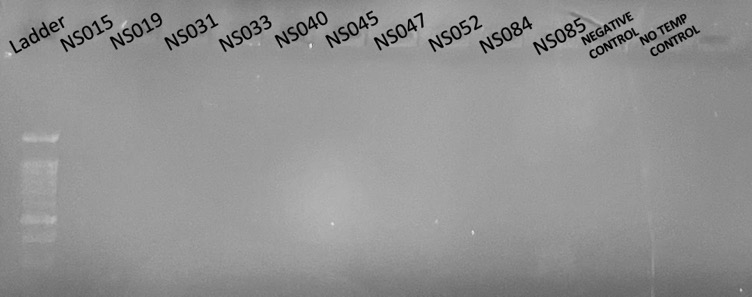
**
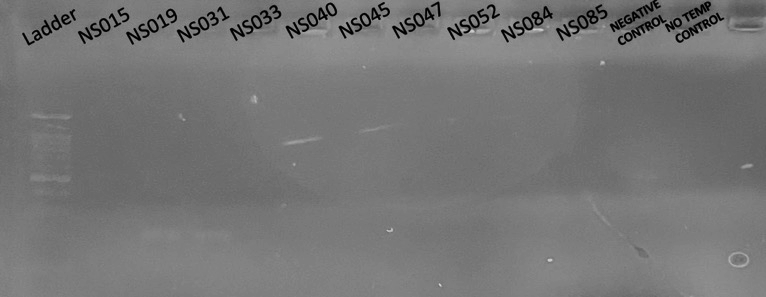


**Supplemental Figure 6.** Agarose gel electrophoresis of qPCR products from no-RT control samples. A: No-RT controls from cDNA synthesis for negative tick samples that underwent qPCR with *EHMT2*-6 primers, with an amplicon size of 207 bp. One sample, NS038, was found to contain residual genomic DNA (box), whereas the rest contained no genomic DNA. B: No-RT controls from cDNA synthesis for negative tick samples that underwent qPCR with *EHMT2*-8 primers, with an amplicon size of 184 bp. NS038 was again found to contain residual genomic DNA (box), whereas the rest contained no genomic DNA. C: No-RT controls from cDNA synthesis for positive tick samples that underwent qPCR with *EHMT2*-6 primers, with an amplicon size of 207 bp. D: No-RT controls from cDNA synthesis for positive tick samples that underwent qPCR with *l13a* primers, with an amplicon size of ~280 bp. The ladder goes from 100bp increments to 1000bp and the top band is 1,500bp.
