# Supplementary material for: Epigenetic responses in Borrelia-infected Ixodes scapularis ticks: Over-expression of euchromatic histone lysine methyltransferase 2 and no change in DNA methylation
Source: PLoS One. 2025 Jun 5;20(6):e0324546. doi: 10.1371/journal.pone.0324546 (PMC12140222; doi:10.1371/journal.pone.0324546)
Supplement: S7 Fig — Agarose gel electrophoresis of negative tick samples NS021, 026, 034, 035, 037, 038, 041, 043, 048. A) Synthesized cDNA from each sample underwent qPCR with EHMT2 6 primers, with an amplicon size of 207 bp. B) Synthesized cDNA from each sample underwent qPCR with EHMT2 8 primers, with an amplicon size of 184 bp. (DOCX) [file pone.0324546.s008.docx]

**Supplemental Figure 7**


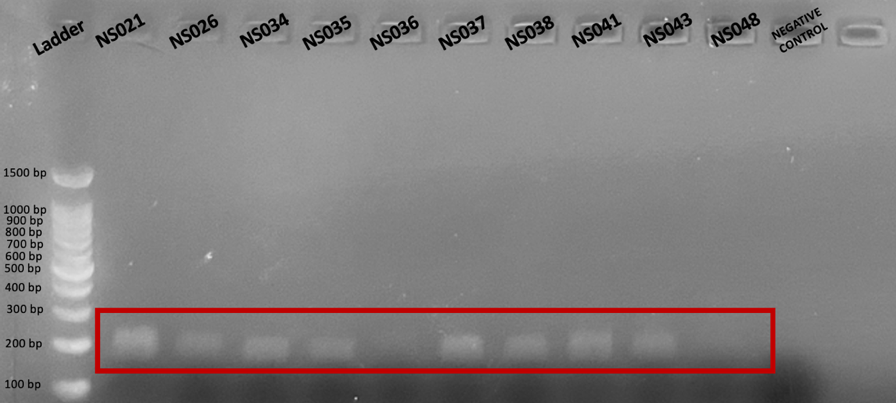

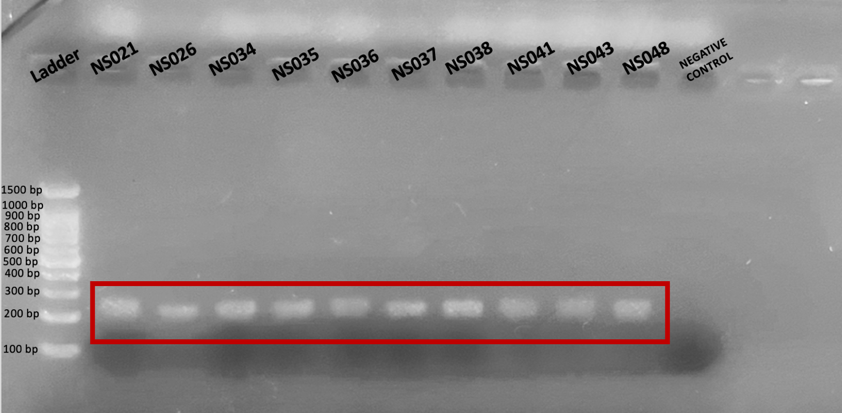


A

B

**Supplemental Figure 7**. Agarose gel electrophoresis of negative tick samples NS021, 026, 034, 035, 036, 037, 038, 041, 043, 048. A: Synthesized cDNA from each sample underwent qPCR with *EHMT2*-6 primers, with an amplicon size of 207 bp. B: Synthesized cDNA from each sample underwent qPCR with *EHMT2*-8 primers, with an amplicon size of 184 bp.
